# Pleiotropic and multicellular roles of lymphotoxin beta receptor in solid tumor immunity and therapeutic targeting

**DOI:** 10.3389/fimmu.2026.1693507

**Published:** 2026-02-04

**Authors:** Anshu, Nair Shantikumar V., Roy Sreeja

**Affiliations:** Amrita Research Center Delhi NCR, Amrita Vishwa Vidyapeetham Faridabad, Faridabad, Haryana, India

**Keywords:** anti-tumor immunity and immunotherapy, lymphotoxin beta receptor, lymphotoxin beta receptor pleiotropy, myeloid reprogramming, non-T cell-based immunotherapy, novel immunotherapeutic approaches, solid tumor immunity, solid tumor microenvironment

## Abstract

Immunotherapy has transformed the treatment landscape of several malignancies, yet solid tumors such as pancreatic ductal adenocarcinoma (PDAC), glioblastoma multiforme (GBM), and triple-negative breast cancer (TNBC) remain largely resistant due to poor immune infiltration, immunosuppressive tumor microenvironments (TMEs) and, the limited success of T cell–centric strategies. The lymphotoxin-beta receptor (LTβR), a member of the tumor necrosis factor (TNF) receptor superfamily, is broadly expressed on stromal, endothelial, and myeloid cells within the TME and signals through both canonical and non-canonical NF-κB pathways. Depending on context and activation mode, LTβR can drive either tumor progression or anti-tumor immunity. While persistent LTβR signaling supports immunosuppressive macrophage phenotypes and promotes tumor growth in hepatocellular carcinoma, preclinical models of colorectal and cervical cancer have demonstrated that LTβR activation induces tertiary lymphoid structures (TLSs), high endothelial venules (HEVs), and immune infiltration, thereby improving responsiveness to immune checkpoint blockade (ICB). This perspective examines in depth the functional duality of LTβR and its emerging therapeutic potential in solid tumors. LTβR agonism has been shown to promote TLS formation and immune activation, whereas antagonistic strategies such as ligand traps may suppress tumor-supportive LTβR signaling in immunosuppressive compartments. Strategically localized LTβR stimulation presents a promising avenue to induce targeted immune reprogramming within the TME. We further explore LTβR’s interactions with key immune subsets—myeloid-derived suppressor cells (MDSCs), dendritic cells (DCs), and tumor-associated macrophages (TAMs)—and its synergy with ICB and CAR T cell therapies. Selective LTβR modulation may reprogram the TME, overcome immunotherapy resistance, and broaden durable responses in refractory solid tumors.

## Graphical Abstract

**Figure:**
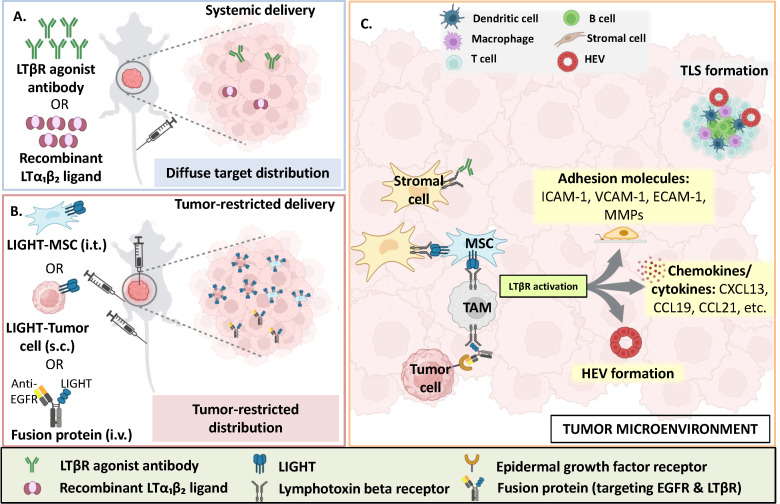
Therapeutic potential of LTβR agonism. **(A)** Systemic delivery of LTβR agonism-based therapeutics. Engagement of LTβR by systemically administered agonistic antibodies or recombinant LTα_1_β_2_ ligand activates LTβR signaling on various cells including stromal, endothelial, and myeloid cells, resulting in broad target distribution and less spatially controlled receptor activation within and beyond the tumor microenvironment (TME). **(B)** Tumor-restricted delivery of LTβR agonism-based therapeutics. In contrast, tumor-targeted strategies such as LIGHT-expressing mesenchymal stromal cells (MSCs), tumor cells, or engineered fusion proteins comprising an anti-epidermal growth factor receptor (EGFR) targeting domain fused to LIGHT enhance intra-tumoral bioavailability and preferentially activate LTβR signaling within the TME. **(C)** LTβR activation within the TME. LTβR agonism induces the expression of chemokines and cytokines including CXCL13, CCL19, and CCL21, as well as adhesion molecules (e.g., ICAM-1, VCAM-1), and promotes high endothelial venule (HEV) differentiation. These coordinated events support tertiary lymphoid structure (TLS) neogenesis, enhance antigen presentation, and facilitate local dendritic cell–T cell cross-talk, collectively promoting anti-tumor immunity. (i.t.: intratumoral; s.c.: subcutaneus; i.v.: intravenous).

## Introduction

Conventional modalities such as surgery, chemotherapy, and radiotherapy remain largely ineffective in advanced solid tumors including Pancreatic ductal adenocarcinoma (PDAC), Triple-negative breast cancer (TNBC, metastatic) and Glioblastoma multiforme (GBM) (1). While immunotherapies, specifically immune checkpoint blockade (ICB), targeting Programmed Cell Death Protein 1 (PD-1), Programmed Death-Ligand 1 (PD-L1), or Cytotoxic T-Lymphocyte–Associated Protein 4 (CTLA-4) has transformed outcomes in melanoma (2) and some lung cancers (3), its efficacy remains limited in tumors such as PDAC and GBM due to immune exclusion, low neoantigen burden, and active immunosuppression by the tumor microenvironment (TME) (4–7). Adoptive cell therapies including Chimeric Antigen Receptor (CAR) T cells (8–10) have shown limited success in solid tumors, primarily due to poor tumor infiltration, T cell exhaustion, and immune escape (11). Cancer vaccines such as GM-CSF–secreting whole-tumor-cell vaccines (e.g., GVAX), peptide-based vaccines, and dendritic cell (DC)–based vaccines have also yielded limited benefit in solid tumors (12–15). While messenger RNA (mRNA)-based neoantigen vaccines and immunomodulatory chemotherapy such as 5-Fluorouracil (5-FU) show promise in preclinical or early clinical settings (16, 17), durable responses remain uncommon. Together, these limitations highlight the urgent need for novel immunotherapeutic strategies beyond conventional T cell–centric approaches to overcome resistance and broaden durable clinical responses.

**Figure 1:**
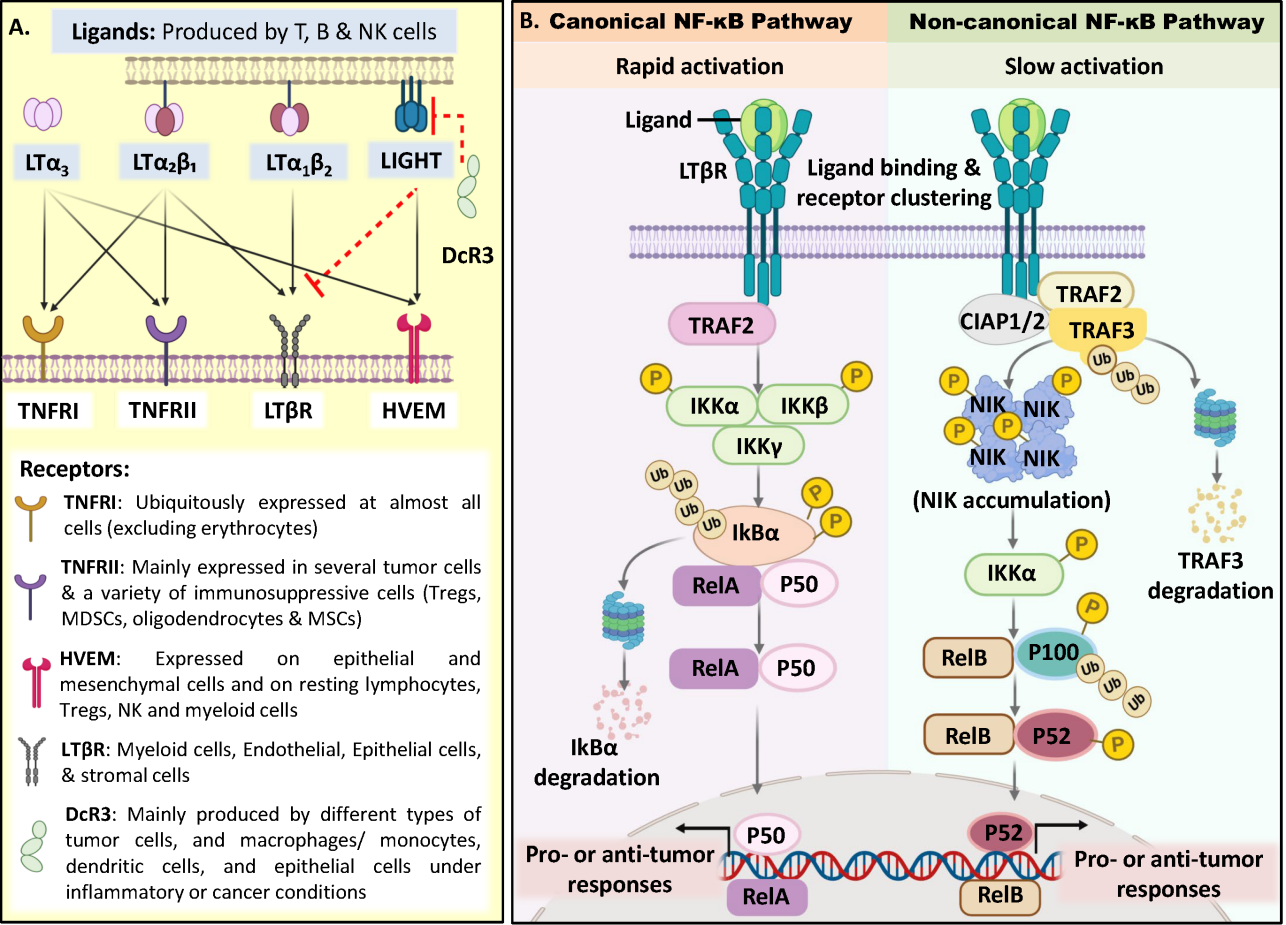
LTβR ligand engagement and downstream NF-κB signaling pathways. **(A)** Ligands and receptors of TNF/lymphotoxin family. The soluble homotrimer LTα_3_ signals through TNFRI, TNFRII and HVEM. Membrane-bound LTα_1_β_2_ is the dominant ligand for LTβR, while LTα_2_β_1_ can engage both LTβR and TNFRI/TNFRII. The homotrimeric ligand LIGHT binds to LTβR as well as HVEM. LTβR signaling is further regulated by DcR3, a soluble decoy receptor that neutralizes LIGHT and limits its interaction with LTβR. Upon activation, LTβR triggers both canonical and non-canonical NF-κB signaling pathways. (LT: Lymphotoxin; TNFR: Tumor Necrosis Factor Receptor; LTβR: Lymphotoxin beta receptor; HVEM: Herpesvirus Entry Mediator; DcR3: Decoy Receptor 3). **(B)** LTβR-mediated NF-κB signaling in solid tumor immunity. Ligand engagement of LTβR induces receptor clustering, recruitment of TNF receptor–associated adaptor proteins (TRAFs), and assembly of the LTβR signaling complex (LTβR-SC). This initiates downstream NF-κB pathways that regulate context-dependent tumor promoting or tumor suppressive roles. Canonical NF-κB activation (rapid) involves TRAF2 recruitment to the cytoplasmic domain of LTβR, resulting in activation of the Inhibitor of κB Kinase (IKK) complex, IKK-mediated phosphorylation and proteasomal degradation of Inhibitor of κB alpha (IκBα), followed by nuclear translocation of RelA/p50 dimers and induction of pro- or anti- tumorigenic responses in the TME. In contrast, the non-canonical NF-κB pathway (slow activation) depends on stabilization of NF-κB–inducing kinase (NIK) and subsequent IKKα-mediated processing of p100 into p52, allowing nuclear translocation of RelB/p52 heterodimers and activation of transcriptional programs governing pro- or anti-tumorigenic outcomes. (123, 124).

Reprogramming non–T cell compartments of the TME, including cancer-associated fibroblasts (CAFs) and tumor-associated macrophages (TAMs), is increasingly recognized as a viable immunotherapeutic strategy. These cells exhibit dual, context-dependent roles in tumor progression and immune activation, that can be therapeutically redirected toward tumor-restrictive phenotypes through CAF modulation and macrophage polarization strategies (18–21). These findings underscore a broader shift toward non–T cell–centric immunotherapeutic interventions. Within this broader landscape, the lymphotoxin-beta receptor (LTβR) emerges as another key immunoregulatory molecule with similarly context-dependent functions. LTβR is primarily expressed on non-hematopoietic cells, particularly stromal (22) and epithelial cells, across both lymphoid and non-lymphoid tissues such as the gut, respiratory tract, and vasculature (23, 24). LTβR is also expressed on tumor cells (22) and on hematopoietic cells, particularly myeloid populations (25), where it supports the homeostasis and function of distinct DC, macrophage, and granulocyte subsets (26–28). Functionally, LTβR is crucial for development and structural organization of lymphoid tissues such as lymph nodes (LN), spleen, and thymus, and regulation of inflammation, adaptive immune responses, and tissue homeostasis (29). LTβR binds to lymphotoxin (LT)αβ heterodimers and LIGHT (TNFSF14), members of the tumor necrosis factor (TNF) superfamily. LTα_1_β_2_ selectively engages LTβR, whereas LIGHT binds both LTβR and herpesvirus entry mediator (HVEM, TNFRSF14) (30, 31). LTβR signaling is modulated by the soluble decoy receptor DcR3 that sequesters LIGHT (32). Downstream, LTβR activates both canonical and non-canonical (alternative) NF-κB pathways, inducing transcription of diverse chemokines and cytokines (31, 33, 34) (**Figure 1A**). The context-dependent expression of LTβR and selective engagement of these pathways underpin its varied roles in tumor progression and anti-tumor immunity, as discussed below.

This review focuses on the dual and context-dependent roles of LTβR signaling in solid tumors, highlighting its capacity to drive both tumor-promoting and anti-tumor immune responses. It emphasizes LTβR’s interactions with myeloid, stromal, and endothelial cells in the TME, and its potential as a therapeutic target. We discuss how selective LTβR modulation can induce tertiary lymphoid structure (TLS) formation, enhance immune cell infiltration, and synergize with existing immunotherapies. Insights from this review aim to guide innovative, TME-focused strategies that improve treatment outcomes in refractory solid cancers.

## LTβR/NF-kB axis: dual role in tumor progression and anti- tumor immunity

The lymphotoxin-beta receptor (LTβR) signaling is initiated by ligand-induced receptor aggregation, leading to the recruitment of TNF receptor-associated factor (TRAF) proteins, assembly of the LTβR signaling complex (LTβR-SC), and activation of NF-κB pathways that mediate apoptosis, inflammation, and tumor progression (35). Rapid LTβR activation recruits TRAF2, triggering Inhibitor of κB kinase (IKK)-mediated phosphorylation and degradation of IκBα, allowing nuclear translocation of RelA/p50 heterodimers to activate the canonical NF-κB pathway. In contrast, slower LTβR activation recruits TRAF3-containing complexes, stabilizing NF-κB-inducing kinase (NIK) and activating IKKα, resulting in nuclear translocation of RelB/p52 dimers driving the non-canonical NF-κB pathway (34, 36–38) (**Figure 1B**). Functionally, canonical NF-κB promotes inflammation and cell survival, whereas non-canonical NF-κB supports lymphoid organ development and immune homeostasis.

Fernandes et al. reviewed the dual role of LTβR in cancer, highlighting the context-dependent LTβR/NF-κB axis in both tumor promotion and anti-tumor immunity (39). Persistent LTβR signaling through the canonical NF-κB pathway has been linked to chronic inflammation and tumor progression (40, 41), whilst non-canonical NF-κB signaling drives stromal differentiation, chemokine secretion and metastasis (42–47). More recently, LTβR has been identified as an immune checkpoint molecule on TAMs, where it sustains immunosuppressive phenotypes and drives ICB resistance via non-canonical NF-κB and Wnt/β-catenin signaling (48). Conversely, LTβR ligation can induce direct CD8^+^ T cell-mediated tumor cytotoxicity via both canonical and non-canonical NF-κB pathways (49). In addition, LTβR activation in tumor cells can indirectly promote anti-tumor responses by upregulating chemokines (50), adhesion molecules (51) and stimulating the formation of specialized blood vessels or high endothelial venules (HEVs). LTβR-induced HEV formation enhances the trafficking of immune cells including ICB-responsive CD8^+^ T cells, and B cells into the tumor that facilitate local immune activation through the development of functional TLS that are organized immune niches within non-lymphoid tissues that closely resemble LN in architecture and function. TLSs provide a localized platform for antigen presentation, lymphocyte priming, and the initiation of adaptive immune responses, thereby enhancing tumor immunosurveillance and promoting anti-tumor immunity (52–54). However, the precise contribution of LTβR-induced NF-κB activation to these processes remains to be fully elucidated.

Taken together, these studies indicate that both rapid (canonical) and slow (non-canonical) LTβR signaling pathways can mediate context-dependent pro- and anti-tumorigenic effects (**Figure 1B**). Notably, prolonged LTβR activation and sustained chronic inflammation have been associated with tumorigenesis (40, 41). Additionally, saturation of LTβR and Fc receptors by excessive ligand binding has been shown to impair receptor clustering and effector function (55). These findings suggest that low-level or transient activation of LTβR, upstream of NF-κB, may be more effective in promoting anti-tumor immunity in solid tumors. Importantly, the mode of LTβR engagement critically influences downstream signaling outcomes. While natural ligands (e.g., LTα_1_β_2_ or LIGHT) activate both canonical and non-canonical NF-κB pathways, they often require co-stimulatory signals or membrane-bound cell contact for full activity (31, 39). In contrast, agonistic LTβR antibodies can induce receptor signaling in a ligand-independent manner (56, 57), enabling more controlled and tunable pathway activation. This property makes agonistic LTβR antibodies particularly attractive as immunotherapeutics for solid tumors, where limited and spatially-restricted receptor clustering may be key to eliciting beneficial immune responses.

## Impact of LTβR-based therapeutics on solid TME

### LTβR agonism

Early studies by Browning and colleagues demonstrated that *in vitro* activation of LTβR using either recombinant LTα_1_β_2_ or an agonistic anti-LTβR monoclonal antibody can induce interferon-γ-dependent cell death in human colorectal adenocarcinoma cells, highlighting the potential of LTβR agonism as a tumoricidal strategy (58, 59). Subsequent investigations extended this role to LTβR-mediated T cell immunity, showing that LTβR signaling can drive tumor regression via immune mechanisms (50, 60, 61). Notably, Lukashev et al. employed a human-specific LTβR agonist antibody (CBE11) and demonstrated that systemic LTβR activation promotes T cell infiltration into the TME in humanized mouse models of colon and cervical cancer, significantly enhancing anti-tumor responses and sensitizing tumors to chemotherapy (62). The recognized role of LTβR in regulating tissue homeostasis, inflammatory responses, and lymphoid tissue organization (34, 63) has increasingly implicated it as a central mediator of TLS formation within solid tumors. Using a murine LTβR agonist (5G1), a recent study demonstrated that systemic LTβR stimulation induces TLS formation in colorectal tumors, characterized by HEVs and enhanced recruitment and interaction of DCs and T cells, ultimately driving tumor-inhibiting immunity (52). Mature TLS containing germinal centers (GCs) composed of follicular helper T (T_FH_) cells, follicular dendritic cells (FDCs), HEVs, and organized T and B cell zones have been associated with improved survival and enhanced ICB responsiveness across solid tumors (64–67), and are enriched in self-renewing progenitor exhausted T cells (T_PEX_) (68), that amplify therapeutic responses (69).

### LTβR- agonism in combination with ICB therapy

T cell- based ICB, particularly PD-1 inhibition, frequently encounters adaptive resistance mechanisms, notably the induction of T cell exhaustion. This occurs through upregulation of molecules such as neurotrophic receptor tyrosine kinase 1 (Ntrk1) in tumors, which limits effector T cell function (70), or through compensatory expression of alternative co-inhibitory receptors like T cell Immunoglobulin and Mucin-domain containing-3 (TIM-3), which promote terminal T cell exhaustion even in the presence of PD-1 blockade (71). To counter this, combinatorial ICB strategies have shown improved efficacy. For instance, dual blockade of PD-1 and CTLA-4 significantly outperforms monotherapy in advanced melanoma patients (72). Similarly, co-targeting PD-1 with other inhibitory receptors such as TIM-3, Lymphocyte Activation Gene-3 (LAG-3), and T cell Immunoreceptor with Ig and ITIM domains (TIGIT) has elicited enhanced anti-tumor responses in clinical trials (73). Although ICB can partially reverse T cell exhaustion and expand responsive subsets such as T_PEX_, a greater pool of T_PEX_ cells also increases the likelihood of their differentiation into terminally exhausted T cells (T_EX_) under persistent antigen exposure, ultimately contributing to treatment refractoriness and limiting long-term efficacy. Given these limitations, targeting immunoregulatory pathways outside of the T cell compartment, such as LTβR expressed on non-lymphoid cells, may alleviate immune pressure on T cells and limit exhaustion, offering a complementary approach to current ICB therapies. Indeed, LTβR agonism has been shown to sensitize tumors to PD-1 blockade and CAR-T cell therapies in murine models (52) and enhance the efficacy of anti-angiogenic and anti–PD-L1 treatments by inducing HEVs (74). In parallel, blockade of upstream T cell intrinsic checkpoint P-selectin glycoprotein ligand-1 (PSGL-1) has been shown to further synergize with PD-1 inhibition to expand ICB-responsive CD8^+^ T cell subsets (75–77). This suggests that combining PSGL-1 blockade with LTβR agonism could synergistically promote the formation of HEVs enriched in stem-like T cells, thereby enhancing ICB responsiveness and improving therapeutic outcomes in immunotherapy-resistant solid tumors.

### LTβR ligands as immunotherapeutic targets

In addition to LTβR agonistic antibodies, LTβR ligands have also been leveraged for tumor targeting. A tumor-specific antibody, LTα fusion protein has been shown to induce TLS within the TME and inhibit both primary tumor growth and metastasis in xenografted and murine models of melanoma (78, 79). Another LTβR ligand, LIGHT (TNFSF14), which binds both LTβR and HVEM has been extensively studied for its anti-tumor potential. Tumor-restricted delivery of the LTβR ligand LIGHT via engineered tumor cells (51), viral vectors or mesenchymal stromal cells (MSCs) (80–82) have led to enhanced T cell infiltration, upregulation of pro-inflammatory cytokines, and in some cases, activation of natural killer (NK) cells (83). Recent studies employing tumor-restricted LIGHT delivery via engineered CAR-T cells or nanoparticles have provided mechanistic insight into how LTβR engagement within the TME induces HEVs and TLS formation, thereby enhancing lymphocyte recruitment and local anti-tumor immunity (84, 85). Moreover, LIGHT-based fusion proteins have been shown to mediate vascular normalization and tumor growth inhibition in an LTβR-dependent manner (86–88). For example, a recombinant fusion protein comprising LIGHT and an anti–epidermal growth factor receptor (EGFR) antibody was engineered to specifically target EGFR-expressing tumor cells, enabling LIGHT engagement with LTβR/HVEM-expressing cells in the TME and facilitating the induction of tumor-reactive T cell immunity in an ICB-resistant fibrosarcoma model (89). Like direct LTβR agonism, LIGHT delivery strategies have also shown enhanced therapeutic efficacy when combined with ICB therapy, demonstrating synergistic anti-tumor effects in preclinical models (80).

Thus, these findings support both systemic and localized LTβR activation as therapeutically effective in promoting anti-tumor immunity via TLS formation in preclinical models (**summarized in graphical abstract**). However, given the broad immunomodulatory functions of LTβR, tumor-directed delivery, analogous to localized LIGHT- based strategies, may preferentially bias receptor engagement toward the TME and reduce the need for widespread systemic activation. Importantly, such approaches do not preclude systemic exposure, and their pharmacokinetics and signaling consequences remain to be defined.

### LTβR blockade in cancer immunotherapy

In solid tumors where LTβR is expressed by immunosuppressive cells such as M2-polarized TAMs, myeloid-derived suppressor cells (MDSCs), and anti-inflammatory stromal cells, LTβR blockade has been shown to reduce tumor burden in preclinical models. In lung adenocarcinoma (LUAD), TAM-specific silencing of LTβR via small interfering RNA (siRNA) enhanced T cell–mediated anti-tumor immunity, reduced MDSC accumulation, and significantly improved the efficacy of ICB therapy (48). Similarly, systemic administration of an LTβR–immunoglobulin fusion protein (LTβR-Ig), which sequesters LTβR ligands and inhibits downstream signaling, resulted in tumor regression by targeting podoplanin-expressing lymph node stromal cells and promoting CD4^+^ T cell–driven anti-tumor responses (90). A recent pan-cancer analysis by Wu et al. demonstrated that LTβR expression correlates with poor prognosis in several tumor types (91). Supporting this, co-culture models revealed that LTβR-mediated cross-talk between stromal cells and ovarian cancer cells promotes tumor proliferation and metastasis (92). These findings suggest that LTβR inhibition in advanced-stage malignancies may improve survival outcomes and augment responsiveness to ICB therapy.

In summary, LTβR exerts dual, context-dependent roles in cancer that vary with tumor type and disease stage (91). Effective clinical translation of LTβR-based immunotherapies will require precise mapping of LTβR expression across TME cell types and their downstream NF-κB signaling dependencies. Persistent or excessive LTβR engagement can drive chronic pro-tumorigenic inflammation and impair receptor clustering through ligand and Fc-receptor saturation (40, 41, 55). Although optimal systemic “low-dose” activation thresholds remain undefined, avoiding excessive LTβR stimulation through tumor-targeted delivery strategies and tumor-specific dose optimization may maximize anti-tumor benefit. Given the heterogeneity of LTβR expression across tumor types, immune subsets, and patient demographics, stratified profiling will be required to guide personalized LTβR-directed immunotherapy.

## Cell-specific expression of LTβR associated with the solid TME

Advanced solid tumors such as PDAC and GBM are classified as immunologically “cold,” “excluded,” or “hot” based on immune infiltration (93–95). Integrated profiling of multiple immune subsets predicts immunotherapy response more accurately than single-cell analyses (96). Tumor infiltration is initiated predominantly by myeloid cells (TAMs, MDSCs, DCs), followed by lymphoid cells (T, B, and NK cells), and is shaped by cytokine- and chemokine-driven recruitment and polarization (97, 98). Given its expression across stromal, endothelial, and myeloid compartments, LTβR-mediated cross-talk is therefore central to therapeutic reprogramming of the TME.

### Non-hematopoietic cells (stromal and endothelial cells)

LTβR signaling plays an essential role in lymph node development through lymphoid tissue organizer (LTo) and lymphoid tissue inducer (LTi) cell interactions leading to chemokine secretion and stromal differentiation (63, 99). In endothelial cells, LTβR promotes HEV formation, facilitating lymphocyte trafficking (63, 99), and tumor studies show that LTβR agonism induces HEVs, TLS-associated chemokines (CXCL13, CCL19, CCL21), and immune infiltration, thereby enhancing ICB efficacy (52).

Stromal cells, particularly CAFs, shape the TME through cytokines, chemokines, extracellular matrix remodeling, and exosomes, fostering immunosuppression and therapy resistance (100). Additional stromal elements like MSCs and tumor endothelial cells (TECs) further regulate immune access and metastatic potential (100). LTβR activation in tumor or lymphatic endothelial cells (LECs) via Treg-derived LTα1β2 promotes angiogenesis and immunosuppressive gene expression through non-canonical NF-κB signaling (101), effects that can be reversed using decoy peptides (nciLT), which reduce Treg/MDSC infiltration and enhance CD8^+^ T cell activity (101).

### Hematopoietic cells (myeloid cells: DCs and macrophages)

In DCs, LTβR promotes maturation, antigen presentation (102), subset specification (103). It also drives TLS formation, chemokine production, and intratumoral T cell activation (52, 104). Within the TME, M1-like (classically activated) TAMs exert anti-tumor functions through Inducible Nitric Oxide Synthase (iNOS), reactive oxygen species (ROS), and pro-inflammatory cytokines (105). Similarly, CD169^+^ macrophages display immunoregulatory and anti-tumor activity, correlating with enhanced cytotoxicity, improved disease prognosis, and increased responsiveness to ICB (106, 107). LTβR signaling regulates their recruitment, antigen capture, and CD8^+^ T cell priming (108).

Conversely, M2-like (alternatively activated) TAMs promote tumor progression via immunosuppressive cytokines, growth factors, and pro-angiogenic mediators (109). In TAMs, LTβR mediates context-specific outcomes, promoting either inflammatory M1-like phenotypes (108), or immunosuppressive M2-like states via noncanonical NF-κB and Wnt/β-catenin signaling, thereby fostering CD8^+^ T cell exhaustion and immune evasion (48). Consequently, LTβR knockdown reverses these immunosuppressive effects and improves immunotherapy outcomes (48). However, CD169^+^ TAMs can also acquire pro-tumorigenic functions in breast cancer through TLS-associated immunosuppression and PD-L1 induction (110–112), highlighting their functional duality in the TME (111).

### Lymphoid cells

LTβR indirectly regulates lymphoid cells through stromal and myeloid intermediates. LTβR signaling in DCs drives Innate lymphoid cell (ILC)- mediated anti-bacterial cytokine production (113), while stromal cells support ILC homeostasis (114). ILC subsets display functional plasticity: NK/ILC1 cells are cytotoxic; ILC2s regulate DC and CD8^+^ T cell recruitment or suppression; and ILC3s modulate tumor growth via cytokines and TLS induction (115). In PDAC, Interleukin (IL)-33^+^ ILCs interact with LTβR^+^ myeloid cells to promote TLS formation and favorable prognosis (116). T cells, as key producers of LTα_1_β_2_, engage LTβR on stromal, myeloid, and ILC populations to drive LN and TLS formation and bidirectional immune modulation (26, 29, 31, 117, 118). Recent studies further show that enforced LTβR expression in CD4^+^ and CD8^+^ T cells enhances effector function and resistance to exhaustion, supporting CAR T cell efficacy (119). Although B cells were traditionally viewed solely as LTβR-ligand producers (29, 117, 118, 120, 121), emerging evidence indicates that murine plasma cells endogenously express LTβR, and enforced LTβR expression in B cells promotes long-lived plasma cell survival and enhanced anti-tumor humoral immunity (122).

## Conclusion

LTβR emerges as a central immunoregulatory node within the solid TME, capable of mediating both tumor-promoting and anti-tumorigenic outcomes depending on cellular context and activation dynamics. Preclinical evidence underscores that judicious activation of LTβR can reprogram immunologically “cold” tumors by inducing TLSs, HEVs, and chemokine-driven lymphocyte trafficking, thereby enhancing responsiveness to ICB and other T cell–based therapies. Conversely, unchecked or chronic LTβR stimulation risks fostering immunosuppressive macrophage phenotypes, stromal remodeling, and tumor progression. These dual roles necessitate the development of precision strategies that balance efficacy with safety. Approaches such as localized delivery of LTβR agonist antibodies, LIGHT-based fusion proteins, or conditionally active bispecific molecules hold promise in achieving spatially controlled receptor clustering and tunable NF-κB activation. Equally important, LTβR blockade in tumors dominated by immunosuppressive myeloid and stromal compartments represents a complementary strategy to reverse resistance and restore anti-tumor immunity. Moving forward, defining the tumor type– and subset-specific expression patterns of LTβR and its downstream signaling dependencies will be critical for the clinical translation of LTβR-targeted therapies. Integration of LTβR modulation with ICB and next-generation vaccines offers a powerful opportunity to overcome T cell–centric immunotherapy limitations and broaden durable responses in refractory solid tumors.

## Data Availability

The original contributions presented in the study are included in the article/supplementary material. Further inquiries can be directed to the corresponding author.
